# A Decision-Making System for Low Carbon Manufacturing

**DOI:** 10.1155/2022/5791880

**Published:** 2022-08-08

**Authors:** Changsong Ma, Tiantong Yuan, Yuzhong Yao, Lixu Wang, Hanyao Gao, Ming Zhang, Liang zhou, Jiaonan Li, Lian Guan

**Affiliations:** ^1^Mianyang Teachers' College, Mianyang 621000, China; ^2^Geely University of China, Beijing, Chengdu 610000, China; ^3^International College, Krirk University, Bangkok 10220, Thailand; ^4^ICO, National Institute of Development Administration, Bangkok 10240, Thailand; ^5^National Human Resources Institute for Service Outsourcing, Tsinghua University, Beijing 1008406, China

## Abstract

Based on the stochastic market demand of products, this paper studies the low-carbon manufacturing decisions making of manufacturing enterprises considering downward substitution and green technology input under the carbon cap-and-trade policy. The results show that the government's carbon trade policy will have a great impact on the production of manufacturing enterprises. Therefore, manufacturing enterprises must attach importance to the constraints of the government's carbon emission reduction policies. In terms of it, there are strategies for manufacturing enterprises such as adjusting the output, trading the carbon emission right, and so on. On this case, green technology input can increase the expected profit of manufacturing enterprises, especially in the case of downward substitution.

## 1. Introduction

Since the industrial revolution, represented by the extensive use of steam engines, fossil fuels such as coal, oil, and natural gas have begun to be widely used as the energy base. While they provide tremendous power and benefits for human production and life, they inevitably produce tremendous environmental side effects [1–3]. Especially in recent years, with the acceleration of human industrialization and urbanization, the combustion of coal, oil, and natural gas has been intensified, the impact on the global environment and climate has been further intensified, the extreme weather has increased, and the trend of global warming has accelerated significantly [1–3]. The Fifth Assessment Report of the IPCC (United Nations Intergovernmental Panel on Climate Change) - Climate Change 2014 pointed out from 2003 to 2012, and the global average surface temperature increased by 0.78°C (0.72–0.85°C) compared with the average temperature from 1850 to 1990 [4]. From 1901 to 2010, the global average sea level has risen by approximately 19 cm. Since the 1950s, more than half of global warming has been caused by greenhouse gas emissions such as carbon dioxide and methane caused by human activities. Currently, the IPCC is in the sixth evaluation cycle. At the invitation of UNFCCC (United Nations Framework Convention on Climate Change), IPCC completed its 6th assessment report on global warming below 1.5 °C and global greenhouse gas emission paths in 2022. The ongoing adverse effects of global climate change have attracted increasing attention from governments and people of all countries. Governments around the world actively take action to formulate policies and set carbon emission reduction targets. Currently, the major carbon emission reduction policies implemented worldwide are as follows: carbon cap policy, carbon emissions tax policy (carbon tax), and carbon emission trading policy (cap-and-trade) [5–7]. Among them, the carbon cap-and-trade policy allows enterprises to trade carbon emission rights freely, which makes carbon emission constraint a kind of “soft restriction,” and makes carbon cap-and-trade become one of the most common carbon emission reduction policies and the policy with the most obvious emission reduction effect [8, 9].

In China's economic system, the industrial sector is the main source of carbon dioxide emissions [9]. As an important part of China's industrial system, the manufacturing industry has become the main force of carbon dioxide emission reduction. In 2001, the total carbon emissions of China's industrial sector were 938 million tons, while in 2011, the total carbon emissions of China's industrial sector exceeded 2.5 billion tons. Carbon dioxide emissions increased by 272% over the decade, with manufacturing accounting for 60% of the total carbon emissions in the industrial sector. The accelerating industrialization process promotes the rapid development of China's economy but also promotes the rapid increase of China's carbon dioxide emissions [10–12].

Moreover, with the increasing awareness of environmental protection, low-carbon consumption has become a trend. Research shows that when green low-carbon products (green products) and ordinary products appear in the market at the same time, although the production cost and price of green products are higher, consumers are not only willing to buy products with low-carbon labels but also willing to buy such products because of the lower carbon emissions in the production and consumption process, which can bring additional utility to consumers. Buying behaviour pays a higher price [13–15]. Therefore, in the face of the government's carbon emission reduction policy and consumer demand, manufacturing enterprises must implement effective low-carbon manufacturing decisions through the production of low-carbon products, the implementation of green low-carbon technology (referred to as “green technology”) for carbon emission purification treatment, and other low-carbon manufacturing methods to obtain additional carbon emission rights [16]. Enterprises can meet the demand of consumers for green life and the policy pressures of low-carbon emission reduction. They can also gain more competitive advantages and higher profits than their competitors [17].

Hence, it is crucial to develop a corporate low-carbon manufacturing decision system based on the carbon emission cap-and-trade policy. It is urgent to help manufacturing companies to reach their carbon reduction goals by developing and designing a low-carbon manufacturing decision system that considers green technology inputs and downward product substitution (i.e., low-carbon products instead of high-carbon products). In addition, it could meet consumer demand and product cost control simultaneously. Enterprises can reap higher expected profits while meeting the requirements of policies toward the green economy. The system can be effectively applied to the occasion of stochastic market demand for the product and solves the strategic problem of enterprise production adjustment and trading of carbon emission rights. This paper proposed a low-carbon manufacturing decision system based on a carbon emission cap-and-trade policy, which is aimed to assist manufacturing enterprises to reach their targets discussed above.

## 2. Literature Review

Benjaafar Li and Daskin [18] took the lead in introducing carbon emission factors into the supply chain system. Through the research, carbon emission factors were found to have an impact on the overall operational decisions of supply chain enterprises. Giraud-Carrier [19] and Gong, Tang et al. [20] studied the operational decision-making process of manufacturing enterprises under three kinds of carbon emission reduction policy constraints and proved that any kind of carbon emission reduction policy constraints will reduce the optimal output of manufacturing enterprises; however, when the environmental pollution is very serious, these carbon emission reduction policies will improve the overall social welfare. Ma, Liu, Zhang, and Wu [21] studied the production decision of carbon-sensitive product manufacturers under the constraints of carbon cap policy and analyzed the impact of the carbon cap policy and product carbon sensitivity on the optimal decision [22]. Ma, He, Luo, and Wu [23] extended the newsboy model. Green technology input was included into the model to study the cross-cycle production decisions of manufacturing enterprises under the constraint of carbon trade policies [24]. He and Ma [25] studied the production decisions of the manufacturing enterprise with two types of products under the carbon trade policies, which obtained the optimal production combination of the manufacturing enterprise and analyzed the impact of carbon trade policies on the optimal decisions of the manufacturing enterprise [26]. Jian, He, Ma, Wu, and Yang [27] studied the pricing decision of competitive and cooperative products in a duopolistic market under the constraint of carbon cap policy, and obtained optimal decisions under the two conditions of competition and cooperation on the basis of extended solution of the Bertrand Game Model [28].

Downward substitution means that when a variety of products with similar performances but different qualities are sold at the same time, if high-quality products have surplus and low-quality products are out of stock, consumers' demand will be satisfied by selling high-quality products with the price of low-quality products. Pentico [29] considered downward substitution between products and used dynamic programming to obtain the optimal production strategy for multiple products. Parlar [30] studied the inventory problem of two alternative products with random demand by using game theory and Nash equilibrium solutions. Chand, Ward, and Weng [31] established a component selection model with downward substitution, and used dynamic programming algorithm to obtain the optimal component inventory combination. Bassok, Anupindi, and Akella [32] studied the inventory problem of single cycle and downward substitution of multiple products, and obtained the optimal production strategy of single cycle products. Pineyro and Viera [33] studied the optimal pricing of new and remanufactured products under nonvolume production conditions. Then, Piñeyro and Viera [34] further studied the batch problem of new and remanufactured products with different consideration of downward substitution in demand flow, and proposed a new algorithm to solve the model. Chen, Chan, and Lee [35] incorporated product substitution on the basis of the Newsboy Model and studied the production decisions of the manufacturing enterprises with two types of products under the constraint of carbon trade policies.

The studies on corporate decisions and downward substitution product models under carbon emission trade have been very rich. However, considering the particularity of carbon emission trade and green technology input in the replacement of low-carbon products to high-carbon products, this paper combines downward substitution with carbon emission trade of low- and high-carbon products, and include green technology input into the model to construct low-carbon manufacturing decisions of manufacturing enterprises considering downward substitution and green technology input under the carbon cap-and-trade regulations.

## 3. Modeling

### 3.1. Basic Model

Without carbon emission policy regulation, *x* and *y* are the random demands of two products, respectively (green product and common product). The *x* and *y* follow the probability density functions of *f*_1_(Δ) and *f*_2_(Δ) for products' demands, respectively. The *p*_*i*_, *c*_*i*_, and *r*_*i*_ (of which *i*=1, 2) are the retail price, production cost of the product, and the opportunity cost of the out-of-stock product per unit. The *c*_*i*_ − *v*_*i*_ is the overproduction cost of the product that exceeds market demand. The *p*_*i*_+*r*_*i*_ − *c*_*i*_ is the out-of-stock cost when the product does not meet market demand. If the output of the manufacturing enterprise is *Q*_1_ and *Q*_2_, the enterprise considers how to produce the two products of downward substitution so as to maximize the expected profit. In all cases, the expected profit of the manufacturing enterprise is as equation (1):

(1) $$\begin{array}{c} \pi^{n}\left(Q_{1},Q_{2}\right)=\left\{\begin{array}{c} \int_{0}^{Q_{1}}\int_{0}^{Q_{2}}\left[p_{1}x+p_{2}y+v_{1}\left(Q_{1}-x\right)+v_{2}\left(Q_{2}-y\right)\right]f\left(x,y\right)\text{d}y\text{d}x,\\ \int_{Q_{1}}^{\infty }\int_{Q_{2}}^{\infty }\left[p_{1}q_{1}+p_{2}Q_{2}-g_{1}\left(x-Q_{1}\right)-g_{2}\left(y-Q_{2}\right)\right]f\left(x,y\right)\text{d}y\text{d}x,\\ \int_{Q_{1}}^{\infty }\int_{0}^{Q_{2}}\left[p_{1}q_{1}+p_{2}y-g_{1}\left(x-Q_{1}\right)+v_{2}\left(Q_{2}-y\right)\right]f\left(x,y\right)\text{d}y\text{d}x,\\ \int_{0}^{Q_{1}}\int_{Q_{2}}^{Q_{1}+Q_{2}-x}\left[p_{1}x+p_{2}y+v_{2}\left(Q_{1}-x-\left(Q_{2}-y\right)\right)\right]f\left(x,y\right)\text{d}y\text{d}x,\\ \int_{0}^{Q_{1}}\int_{Q_{1}+Q_{2}-x}^{\infty }\left[p_{1}x+p_{2}\left(Q_{1}+Q_{2}-x\right)-g_{2}\left(y-\left(Q_{1}+Q_{2}-x\right)\right)\right]f\left(x,y\right)\text{d}y\text{d}x-c_{1}Q_{1}-c_{2}Q_{2}. \end{array}\right. \end{array}$$

The above function indicates as follows:

When the demand for both products is less than the respective output, the profit of the manufacturing enterprise is the total revenue minus the sum of production costs and the loss cost of unsold products of the two products.

When the demand for both products is higher than the respective output, the profit of the manufacturing enterprise is the total revenue minus the sum of total production cost and out-of-stock cost of the two products.

When green product is out of stock and common product is surplus, since common product cannot replace green product, the profit of the manufacturing enterprise is the total revenue minus the total production cost, the out-of-stock cost of green product and the loss cost of the unsold common product.

When green product is surplus and common product is out of stock, and the out-of-stock quantity of the common product is less than the remaining green product, the manufacturing enterprise will use the remaining green product to meet the out-of-stock demand of the common product, and the profit of the manufacturing enterprise is the total revenue minus the total production cost and loss cost of the unsold green product after downward substitution. In (1), ∫_0_^*Q*_1_^∫_*Q*_2__^*Q*_1_+*Q*_2_−*x*^[(*Q*_1_ − *x* − (*Q*_2_ − *y*))]*f*(*x*, *y*)d*y*d*x*represents the expectation that green products will remain in stock after downward substitution.

When green product is surplus and common product is out of stock, and the out-of-stock quantity of ordinary product is more than the remaining green product, the manufacturing enterprise will use the remaining green product to meet the out-of-stock demand of the common product. At this time, the profit of the manufacturing enterprise is the total revenue minus total production cost and the out-of-stock cost after downward substitution. In equation (1), ∫_0_^*Q*_1_^∫_*Q*_1_+*Q*_2_−*x*_^*∞*^[(*y* − (*Q*_1_+*Q*_2_ − *x*))]*f*(*x*, *y*)d*y*d*x* represents the expectation that green products are still out of stock after downward substitution.

The expected profit of the manufacturing enterprise is as (2):

(2) $$\begin{array}{c} \pi^{n}\left(Q_{1},Q_{2}\right)=\int_{0}^{Q_{1}}\int_{0}^{Q_{2}}\left[p_{1}x+p_{2}y+v_{1}\left(Q_{1}-x\right)+v_{2}\left(Q_{2}-y\right)\right]f\left(x,y\right)\text{d}y\text{d}x\\ +\int_{Q_{1}}^{\infty }\int_{Q_{2}}^{\infty }\left[p_{1}Q_{1}+p_{2}Q_{2}-g_{1}\left(x-Q_{1}\right)-g_{2}\left(y-Q_{2}\right)\right]f\left(x,y\right)\text{d}y\text{d}x\\ +\int_{Q_{1}}^{\infty }\int_{0}^{Q_{2}}\left[p_{1}Q_{1}+p_{2}y-g_{1}\left(x-Q_{1}\right)+v_{2}\left(Q_{2}-y\right)\right]f\left(x,y\right)\text{d}y\text{d}x\\ +\int_{0}^{Q_{1}}\int_{Q_{2}}^{Q_{1}+Q_{2}-x}\left[p_{1}x+p_{2}y+v_{1}\left(Q_{1}-x-\left(Q_{2}-y\right)\right)\right]f\left(x,y\right)\text{d}y\text{d}x\\ +\int_{0}^{Q_{1}}\int_{Q_{1}+Q_{2}-x}^{\infty }\left[p_{1}x+p_{2}\left(Q_{1}+Q_{2}-x\right)-g_{2}\left(y-\left(Q_{1}+Q_{2}-x\right)\right)\right]\left(x,y\right)\text{d}y\text{d}x-c_{1}Q_{1}-c_{2}Q_{2}. \end{array}$$

It is obtained by solving the first partial derivatives of *Q*_1_ and *Q*_2_ of (1), respectively:

(3) $$\begin{array}{c} \frac{\partial \pi^{n}\left(Q_{1},Q_{2}\right)}{\partial Q_{1}}=\left(r_{2}-r_{1}\right)F_{1}\left(Q_{1}\right)+\left(v_{1}-r_{2}\right)\int_{0}^{Q_{1}}\int_{0}^{Q_{1}+Q_{2}-x}f\left(x,y\right)\text{d}y\text{d}x+r_{1}-c_{1},\\ \frac{\partial \pi^{n}\left(Q_{1},Q_{2}\right)}{\partial Q_{2}}=\left(v_{1}-r_{2}\right)\left[\int_{0}^{Q_{1}}\int_{0}^{Q_{1}+Q_{2}-x}f\left(x,y\right)\text{d}y\text{d}x-F\left(Q_{1},Q_{2}\right)\right]+\left(v_{2}-r_{2}\right)F_{2}\left(Q_{2}\right)+r_{2}-c_{2}. \end{array}$$

It is obtained by solving the second partial derivatives of *Q*_1_ and *Q*_2_ of (1) respectively:

(4) $$\begin{array}{c} \frac{\partial^{2}\;\pi^{n}\left(Q_{1},Q_{2}\right)}{\partial Q_{1}^{2}}=\left(r_{2}-r_{1}\right)f_{1}\left(Q_{1}\right)+\left(v_{1}-r_{2}\right)\left[\int_{0}^{Q_{1}}f\left(x,Q_{1}+Q_{2}-x\right)\text{d}x+\int_{0}^{Q_{2}}f\left(Q_{1},y\right)\text{d}y\right]<0,\\ \frac{\partial^{2}\;\pi^{n}\left(Q_{1},Q_{2}\right)}{\partial Q_{2}^{2}}=\left(v_{1}-r_{2}\right)\left[\int_{0}^{Q_{1}}f\left(x,Q_{1}+Q_{2}-x\right)\text{d}x-\int_{0}^{Q_{1}}f\left(x,Q_{2}\right)\text{d}x\right]+\left(v_{2}-r_{2}\right)f_{2}\left(Q_{2}\right)\leq \left(v_{1}-r_{2}\right)\left[\int_{0}^{Q_{1}}f\left(x,Q_{1}+Q_{2}-x\right)dx+\int_{Q_{1}}^{\infty }f\left(x,Q_{2}\right)\text{d}x\right]<0. \end{array}$$

The second mixed partial derivatives of *Q*_1_ and *Q*_2_ are solved as

(5) $$\begin{array}{c} \frac{\partial^{2}\;\pi^{n}\left(Q_{1},Q_{2}\right)}{\partial Q_{1}\partial Q_{2}}=\frac{\partial^{2}\;\pi^{n}\left(Q_{1},Q_{2}\right)}{\partial Q_{2}\partial Q_{1}}\\ =\left(v_{1}-r_{2}\right)\int_{0}^{Q_{1}}f\left(x,Q_{1}+Q_{2}-x\right)\text{d}x<0. \end{array}$$

Thus,

(6) $$\begin{array}{c} \left|\begin{array}{cc} \frac{\partial^{2}\;\pi^{n}\left(Q_{1},Q_{2}\right)}{\partial Q_{1}^{2}} & \frac{\partial^{2}\;\pi^{n}\left(Q_{1},Q_{2}\right)}{\partial Q_{1}\partial Q_{2}}\\ \frac{\partial^{2}\;\pi^{n}\left(Q_{1},Q_{2}\right)}{\partial Q_{2}\partial Q_{1}} & \frac{\partial^{2}\;\pi^{n}\left(Q_{1},Q_{2}\right)}{\partial Q_{2}^{2}} \end{array}\right|=\frac{\partial^{2}\;\pi^{n}\left(Q_{1},q_{2}\right)}{\partial Q_{1}^{2}}\frac{\partial^{2}\;\pi^{n}\left(Q_{1},q_{2}\right)}{\partial q_{2}^{2}}-\frac{\partial^{2}\;\pi^{n}\left(Q_{1},q_{2}\right)}{\partial Q_{1}\partial q_{2}}\frac{\partial^{2}\;\pi^{n}\left(Q_{1},q_{2}\right)}{\partial q_{2}\partial Q_{1}}>0. \end{array}$$

Therefore,  *π*^*n*^(*Q*_1_, *Q*_2_) is the concave function of *Q*_1_ and *Q*_2_, let *∂π*^*n*^(*Q*_1_, *Q*_2_)/*∂Q*_1_=0 and *∂π*^*n*^(*Q*_1_, *Q*_2_)/*∂Q*_2_=0; thus,

(7) $$\begin{array}{c} F_{1}\left(Q_{1}\right)+\frac{r_{2}-v_{1}}{r_{1}-r_{2}}\int_{0}^{Q_{1}}\int_{0}^{Q_{1}+Q_{2}-x}f\left(x,y\right)\text{d}y\text{d}x=\frac{r_{1}-c_{1}}{r_{1}-r_{2}},\\ F_{2}\left(Q_{2}\right)+\frac{r_{2}-v_{1}}{r_{2}-v_{2}}\left[\int_{0}^{Q_{1}}\int_{0}^{Q_{1}+Q_{2}-x}f\left(x,y\right)\text{d}y\text{d}x-F\left(Q_{1},Q_{2}\right)\right]=\frac{r_{2}-c_{2}}{r_{2}-v_{2}}. \end{array}$$

The above model shows that the unique optimal manufacturing decision for manufacturing enterprises exists without carbon emission policy regulation.

Under the government carbon cap regulation, there is a fixed maximum carbon emission *K*, in which the carbon emission produced by manufacturing enterprises in production activities cannot exceed *K*. Therefore, the expected profit of the manufacturing enterprise is as (8) and (9):

(8) $$\begin{array}{c} \max\pi^{a}\left(Q_{1},Q_{2}\right)=\pi^{n}\left(Q_{1},Q_{2}\right), \end{array}$$

(9) $$\begin{array}{c} s.t\;k_{1}Q_{1}+k_{2}Q_{2}\leq K. \end{array}$$

The constraint condition means that the total carbon emissions of manufacturing enterprises in production activities must not exceed the carbon cap set by the government. By discussing the optimal manufacturing decision of the manufacturing enterprise in this case, the expected profit increment of the manufacturing enterprise brought by the unit carbon emission is

(10) $$\begin{array}{c} \Delta \pi^{n}\left(Q_{1},Q_{2}\right)=\pi^{n}{\left(Q_{1},Q_{2}\right)}_{Qi}^{\prime }\\ =\partial \pi^{n}\left(Q_{1},Q_{2}\right). \end{array}$$

Therefore, the expected profit growth of the manufacturing enterprise brought by the unit carbon emissions is *θ*_1_(*Q*_1_)=(1/*k*_1_)(*∂π*^*n*^(*Q*_1_, *Q*_2_)/*∂Q*_1_) and *θ*_2_(*Q*_2_)=(1/*k*_2_)(*∂π*^*n*^(*Q*_1_, *Q*_2_)/*∂Q*_2_).

When *θ*_*i*_(*Q*_*i*_) > 0, manufacturing enterprises could increase the expected profit by increasing production.

When *θ*_*i*_(*Q*_*i*_) < 0, manufacturing enterprises could not increase the expected profit by increasing production.

When *θ*_*i*_(*Q*_*i*_)=0, the output of the product at this present can maximize the expected profit of the manufacturing enterprise.

By discussing the optimal manufacturing decision of the enterprise in this case, the propositions are obtained

**Proposition 1 .**

Under the government carbon cap regulation, considering downward substitution, when the manufacturing enterprise meets *θ*_1_(*Q*_1_^*a*^)=*θ*_2_(*Q*_2_^*a*^), optimal output is *Q*_1_^*a*^ ≤ *Q*_1_^*∗*^, *Q*_2_^*a*^ ≤ *Q*_2_^*∗*^ under the carbon cap.

**Proof**

Let *φ* ≥ 0, Equations (11)–(14) can be obtained from the constraint condition:

(11) $$\begin{array}{c} k_{1}Q_{1}+\;k_{2}Q_{2}-K\leq 0, \end{array}$$

(12) $$\begin{array}{c} \varphi \left(k_{1}Q_{1}+\;k_{2}Q_{2}-K\right)=0, \end{array}$$

(13) $$\begin{array}{c} \left(r_{2}-r_{1}\right)F_{1}\left(Q_{1}\right)+\left(v_{1}-r_{2}\right)\int_{0}^{Q_{1}}\int_{0}^{Q_{1}+Q_{2}-x}f\left(x,y\right)\text{d}y\text{d}x+r_{1}-c_{1}-\lambda_{1}k_{1}=0, \end{array}$$

(14) $$\begin{array}{c} \left(v_{1}-r_{2}\right)\left[\int_{0}^{Q_{1}}\int_{0}^{Q_{1}+Q_{2}-x}f\left(x,y\right)\text{d}y\text{d}x-F\left(Q_{1},Q_{2}\right)\right]+\left(v_{2}-r_{2}\right)F_{2}\left(Q_{2}\right)+r_{2}-c_{2}-\lambda_{1}k_{2}=0. \end{array}$$

When *φ*=0, it is obtained that *∂π*^*n*^(*Q*_1_, *Q*_2_)/*∂Q*_1_=0, (*∂π*^*n*^(*Q*_1_, *Q*_2_)/*∂Q*_2_)=0 from (13) and (14). Therefore, *Q*_1_^*∗*^=*Q*_1_^*a*^, *Q*_2_^*a*^=*Q*_2_^*∗*^ is obtained, which *k*_1_*Q*_1_^*∗*^+*k*_2_*Q*_2_^*∗*^ ≤ *K*.

When *φ* > 0, it is obtained from (13) and (14) that

(15) $$\begin{array}{c} \frac{\partial \pi^{n}\left(Q_{1},Q_{2}\right)}{\partial Q_{1}}=\left(r_{2}-r_{1}\right)F_{1}\left(Q_{1}\right)+\left(v_{1}-r_{2}\right)\int_{0}^{Q_{1}}\int_{0}^{Q_{1}+Q_{2}-x}f\left(x,y\right)\text{d}y\text{d}x+r_{1}-c_{1}=\lambda_{1}k_{1}>0,\\ \frac{\partial \pi^{n}\left(Q_{1},Q_{2}\right)}{\partial Q_{2}}=\left(v_{1}-r_{2}\right)\left[\int_{0}^{Q_{1}}\int_{0}^{Q_{1}+Q_{2}-x}f\left(x,y\right)\text{d}y\text{d}x-F\left(Q_{1},Q_{2}\right)\right]+\left(v_{2}-r_{2}\right)F_{2}\left(Q_{2}\right)+r_{2}-c_{2}=\lambda_{1}k_{2}>0. \end{array}$$

Therefore, *Q*_1_^*a*^ < *Q*_1_^*∗*^, *Q*_2_^*a*^ < *Q*_2_^*∗*^ is obtained:

(16) $$\begin{array}{c} \frac{1}{k_{1}}\left[\left(r_{2}-r_{1}\right)F_{1}\left(Q_{1}\right)+\left(v_{1}-r_{2}\right)\int_{0}^{Q_{1}}\int_{0}^{Q_{1}+Q_{2}-x}f\left(x,y\right)\text{d}y\text{d}x+r_{1}-c_{1}\right]=\frac{1}{k_{2}}\left[\left(v_{1}-r_{2}\right)\left[\int_{0}^{Q_{1}}\int_{0}^{Q_{1}+Q_{2}-x}f\left(x,y\right)\text{d}y\text{d}x-F\left(Q_{1},Q_{2}\right)+\left(v_{2}-r_{2}\right)F_{2}\left(Q_{2}\right)+r_{2}-c_{2}\right]\right]. \end{array}$$

Hence, the proof is justified.

Proposition 1 indicates that under the government`s carbon cap regulation, manufacturing enterprises meet *θ*_1_(*Q*_1_^*a*^)=*θ*_2_(*Q*_2_^*a*^).

When *θ*_1_(*Q*_1_^*a*^) > *θ*_2_(*Q*_2_^*a*^), manufacturing enterprises can produce more green products to achieve higher expected profits.

When *θ*_1_(*Q*_1_^*a*^) < *θ*_2_(*Q*_2_^*a*^), manufacturing enterprises can produce more common products to achieve higher expected profits.

On the other hand, the optimal output of ordinary products and green products under the carbon cap will never be higher than that without the carbon cap. This means that in order to maintain a good environment, manufacturing enterprises must meet the government's carbon emission policy and pay for certain economic price.

### 3.2. No Green Technology Input under Carbon Trade Policy

Let *E* be the trading volume of carbon emissions made by the enterprises from external markets. Therefore, (17) and (18) are the expected profit after considering downward substitution and carbon emission rights trade under the carbon trade policy:

(17) $$\begin{array}{c} \max\;\pi^{e}\left(Q_{1},Q_{2}\right)=\;\pi^{n}\left(Q_{1},Q_{2}\right)-wE, \end{array}$$

(18) $$\begin{array}{c} s.t\;k_{1}Q_{1}+\;k_{2}Q_{2}=K+E. \end{array}$$

The constraint condition means that the amount of carbon emissions of manufacturing enterprise is equal to the sum of the initial carbon emission cap set by the government and the carbon emissions trade in the external market.

When *E* > 0 , manufacturing enterprise will purchase carbon rights from external market.

When *E*=0 , manufacturing enterprise will not trade carbon rights in external market.

When *E* < 0, manufacturing enterprise will sell inexhaustible carbon rights in external market.

The expected profit growth of manufacturing enterprise brought by unit carbon rights is *θ*_1_(*Q*_1_)=(1/*k*_1_)(*∂π*^*n*^(*Q*_1_, *Q*_2_)/*∂Q*_1_) and *θ*_2_(*Q*_2_)=(1/*k*_2_)(*∂π*^*n*^(*Q*_1_, *Q*_2_)/*∂Q*_2_),

By discussing the optimal manufacturing decision of the enterprise in this case, the following propositions are obtained:

**Proposition 2 .**

Under the regulation of carbon trade policy, considering downward substitution, there is an optimal manufacturing decision that maximizes the enterprise's expected profit and meets *θ*_1_(*Q*_1_^*e*^)=*θ*_2_(*Q*_2_^*e*^)=*w*.

**Proof**

*E*=*k*_1_*Q*_1_+ *k*_2_*Q*_2_ − *K* is obtained from (18); therefore, the expected profit of the manufacturing enterprise is

(19) $$\begin{array}{c} \pi^{e}\left(Q_{1},Q_{2}\right)=\int_{0}^{Q_{1}}\int_{0}^{Q_{2}}\left[p_{1}x+p_{2}y+v_{1}\left(Q_{1}-x\right)+v_{2}\left(Q_{2}-y\right)\right]f\left(x,y\right)\text{d}y\text{d}x\\ +\int_{Q_{1}}^{\infty }\int_{Q_{2}}^{\infty }\left[p_{1}Q_{1}+p_{2}Q_{2}-g_{1}\left(x-Q_{1}\right)-g_{2}\left(y-Q_{2}\right)\right]f\left(x,y\right)\text{d}y\text{d}x+\int_{Q_{1}}^{\infty }\int_{0}^{Q_{2}}\left[p_{1}Q_{1}+p_{2}y-g_{1}\left(x-Q_{1}\right)+v_{2}\left(Q_{2}-y\right)\right]f\left(x,y\right)\text{d}y\text{d}x\\ +\int_{0}^{Q_{1}}\int_{Q_{2}}^{Q_{1}+Q_{2}-x}\left[p_{1}x+p_{2}y+v_{1}\left(Q_{1}-x-\left(Q_{2}-y\right)\right)\right]f\left(x,y\right)\text{d}y\text{d}x+\int_{0}^{Q_{1}}\int_{Q_{1}+Q_{2}-x}^{\infty }\left[p_{1}x+p_{2}\left(Q_{1}+Q_{2}-x\right)-g_{2}\left(y-\left(Q_{1}+Q_{2}-x\right)\right)\right]f\left(x,y\right)\text{d}y\text{d}x-c_{1}Q_{1}-c_{2}Q_{2}-w\left(k_{1}Q_{1}+\;k_{2}Q_{2}-K\right). \end{array}$$

It is obtained by solving the first partial derivatives of *Q*_1_ and *Q*_2_, respectively:

(20) $$\begin{array}{c} \frac{\partial \pi^{e}\left(Q_{1},Q_{2}\right)}{\partial Q_{1}}=\left(r_{2}-r_{1}\right)F_{1}\left(Q_{1}\right)+\left(v_{1}-r_{2}\right)\int_{0}^{Q_{1}}\int_{0}^{Q_{1}+Q_{2}-x}f\left(x,y\right)\text{d}y\text{d}x+r_{1}-c_{1}-wk_{1},\\ \frac{\partial \pi^{e}\left(Q_{1},Q_{2}\right)}{\partial Q_{2}}=\left(v_{1}-r_{2}\right)\left[\int_{0}^{Q_{1}}\int_{0}^{Q_{1}+Q_{2}-x}f\left(x,y\right)\text{d}y\text{d}x-F\left(Q_{1},Q_{2}\right)\right]+\left(v_{2}-r_{2}\right)F_{2}\left(Q_{2}\right)+r_{2}-c_{2}-wk_{2}. \end{array}$$

It is obtained by solving the second partial derivatives of *Q*_1_ and *Q*_2_, respectively:

(21) $$\begin{array}{c} \frac{\partial^{2}\pi^{e}\left(Q_{1},Q_{2}\right)}{\partial Q_{1}{}^{2}}=\left(r_{2}-r_{1}\right)f_{1}\left(Q_{1}\right)+\left(v_{1}-r_{2}\right)\left[\int_{0}^{Q_{1}}f\left(x,Q_{1}+Q_{2}-x\right)\text{d}x+\int_{0}^{Q_{2}}f\left(Q_{1},y\right)\text{d}y\right]<0,\\ \frac{\partial^{2}\pi^{e}\left(Q_{1},Q_{2}\right)}{\partial Q_{2}{}^{2}}=\left(v_{1}-r_{2}\right)\left[\int_{0}^{Q_{1}}f\left(x,Q_{1}+Q_{2}-x\right)\text{d}x-\int_{0}^{Q_{1}}f\left(x,Q_{2}\right)\text{d}x\right]+\left(v_{2}-r_{2}\right)f_{2}\left(Q_{2}\right)\leq \left(v_{1}-r_{2}\right)\left[\int_{0}^{Q_{1}}f\left(x,Q_{1}+Q_{2}-x\right)\text{d}x+\int_{Q_{1}}^{\infty }f\left(x,Q_{2}\right)\text{d}x\right]<0. \end{array}$$

The second mixed partial derivatives of *Q*_1_ and *Q*_2_ are solved as

(22) $$\begin{array}{c} \frac{\partial^{2}\pi^{e}\left(Q_{1},Q_{2}\right)}{\partial Q_{1}\partial Q_{2}}=\frac{\partial^{2}\pi^{e}\left(Q_{1},Q_{2}\right)}{\partial Q_{2}\partial Q_{1}}\\ =\left(v_{1}-r_{2}\right)\int_{0}^{Q_{1}}f\left(x,Q_{1}+Q_{2}-x\right)\text{d}x<0. \end{array}$$

Therefore,

(23) $$\begin{array}{c} \left|\begin{array}{cc} \frac{\partial^{2}\pi^{e}\left(Q_{1},Q_{2}\right)}{\partial Q_{1}^{2}} & \frac{\partial^{2}\pi^{e}\left(Q_{1},Q_{2}\right)}{\partial Q_{1}\partial Q_{2}}\\ \frac{\partial^{2}\pi^{e}\left(Q_{1},Q_{2}\right)}{\partial Q_{2}\partial Q_{1}} & \frac{\partial^{2}\pi^{e}\left(Q_{1},Q_{2}\right)}{\partial Q_{2}^{2}} \end{array}\right|=\frac{\partial^{2}\pi^{e}\left(Q_{1},Q_{2}\right)}{\partial Q_{1}^{2}}\frac{\partial^{2}\pi^{e}\left(Q_{1},Q_{2}\right)}{\partial Q_{2}^{2}}-\frac{\partial^{2}\pi^{e}\left(Q_{1},Q_{2}\right)}{\partial Q_{1}\partial Q_{2}}\frac{\partial^{2}\pi^{e}\left(Q_{1},Q_{2}\right)}{\partial Q_{2}\partial Q_{1}}>0. \end{array}$$

Therefore, *π*^*e*^(*Q*_1_, *Q*_2_) is the concave function of *Q*_1_ and *Q*_2_, let *∂π*^*e*^(*Q*_1_, *Q*_2_)/*∂Q*_1_=0;thus,

(24) $$\begin{array}{c} \left(r_{2}-r_{1}\right)F_{1}\left(Q_{1}\right)+\left(v_{1}-r_{2}\right)\int_{0}^{Q_{1}}\int_{0}^{Q_{1}+Q_{2}-x}f\left(x,y\right)\text{d}y\text{d}x+r_{1}-c_{1}-wk_{1}=0. \end{array}$$

*θ*_1_(*Q*_1_^*e*^)=*w* is obtained. Let *∂π*^*e*^(*Q*_1_, *Q*_2_)/*∂Q*_2_=0; thus,

(25) $$\begin{array}{c} \left(v_{1}-r_{2}\right)\left[\int_{0}^{Q_{1}}\int_{0}^{Q_{1}+Q_{2}-x}f\left(x,y\right)\text{d}y\text{d}x-F\left(Q_{1},Q_{2}\right)\right]+\left(v_{2}-r_{2}\right)F_{2}\left(Q_{2}\right)+r_{2}-c_{2}-wk_{2}=0, \end{array}$$

*Q*_2_^*e*^=*w* is obtained.

Hence, the proof is justified.

Proposition 2 shows that under the regulation of carbon trade policy and considering downward substitution, the optimal manufacturing decision of the manufacturing enterprise meets the conditions of *θ*_1_(*Q*_1_^*e*^)=*θ*_2_(*Q*_2_^*e*^); otherwise, manufacturing enterprise can produce more green or common products to achieve higher expected profits.

In this case, the manufacturing enterprise`s maximum expected profit is as (26):

(26) $$\begin{array}{c} \pi^{*e}\left(Q_{1}^{e},Q_{2}^{e}\right)=\pi^{*n}\left(Q_{1}^{e},Q_{2}^{e}\right)-w\left(k_{1}Q_{1}^{e}+k_{2}Q_{2}^{e}-K\right). \end{array}$$

### 3.3. Considering Green Technology Input under Carbon Trade Policy

More and more enterprises have realized that relying on green technology such as technological innovation can improve energy utilization and reduce carbon dioxide emissions, thus bringing new profit growth points for themselves. If *T* is the level of green technology input, manufacturing enterprises conduct green technology input to ordinary products; in this case, the expected profit is as (27) and (28):

(27) $$\begin{array}{c} \pi^{c}\left(Q_{1},Q_{2},T\right)=\int_{0}^{Q_{1}}\int_{0}^{Q_{2}}\left[p_{1}x+p_{2}y+v_{1}\left(Q_{1}-x\right)+v_{2}\left(Q_{2}-y\right)\right]f\left(x,y\right)\text{d}y\text{d}x\\ +\int_{Q_{1}}^{\infty }\int_{0}^{Q_{2}}\left[p_{1}Q_{1}+p_{2}y-g_{1}\left(x-Q_{1}\right)+v_{2}\left(Q_{2}-y\right)\right]f\left(x,y\right)\text{d}y\text{d}x\\ +\int_{0}^{Q_{1}}\int_{Q_{2}}^{Q_{1}+Q_{2}-x}\left[p_{1}x+p_{2}y+v_{1}\left(Q_{1}-x-\left(Q_{2}-y\right)\right)\right]f\left(x,y\right)\text{d}y\text{d}x\\ +\int_{0}^{Q_{1}}\int_{Q_{1}+Q_{2}-x}^{\infty }\left[p_{1}x+p_{2}\left(Q_{1}+Q_{2}-x\right)-g_{2}\left(y-\left(Q_{1}+Q_{2}-x\right)\right)\right]f\left(x,y\right)\text{d}y\text{d}x-c_{1}Q_{1}-c_{2}Q_{2}-c\left(T\right), \end{array}$$

(28) $$\begin{array}{c} \text{s.t }k_{1}Q_{1}+\;\left(1-T\right)k_{2}Q_{2}=K+E. \end{array}$$

Constraint condition means that in this case, the total carbon emissions of manufacturing enterprise after green technology input still must be equal to the sum of the government's initial carbon cap and the carbon emissions trade in the external carbon trade market.

By discussing the optimal manufacturing decision of the manufacturing enterprise in this case, the following propositions are obtained:

**Proposition 3 .**

under the regulation of carbon trade policy, considering downward substitution, manufacturing enterprises carry out green technology input, there is an optimal manufacturing decision that maximizes enterprise`s expected profit, and *θ*_2_(*Q*_2_^*c*^)=(1 − *T*)*w*.

**Proof**

From (27), *E*=*k*_1_*Q*_1_+ (1 − *T*)*k*_2_*Q*_2_ − *K* is obtained. From (27) and (28), it is obtained that

(29) $$\begin{array}{c} \pi^{c}\left(Q_{1},Q_{2},T\right)=\int_{0}^{Q_{1}}\int_{0}^{Q_{2}}\left[p_{1}x+p_{2}y+v_{1}\left(Q_{1}-x\right)+v_{2}\left(Q_{2}-y\right)\right]f\left(x,y\right)\text{d}y\text{d}x\\ +\int_{Q_{1}}^{\infty }\int_{Q_{2}}^{\infty }\left[p_{1}Q_{1}+p_{2}Q_{2}-g_{1}\left(x-Q_{1}\right)-g_{2}\left(x-Q_{2}\right)\right]f\left(x,y\right)dy\;dx\\ +\int_{Q_{1}}^{\infty }\int_{0}^{Q_{2}}\left[p_{1}Q_{1}+p_{2}y-g_{1}\left(x-Q_{1}\right)-v_{2}\left(Q_{2}-y\right)\right]f\left(x,y\right)dy\;dx\\ +\int_{0}^{Q_{1}}\int_{Q_{2}}^{Q_{1}+Q_{2}-x}\left[p_{1}x+p_{2}y-v_{1}\left(Q_{1}-x-\left(Q_{2}-y\right)\right)\right]f\left(x,y\right)dy\;dx\\ +\int_{0}^{Q_{1}}\int_{Q_{1}+Q_{2}-x}^{\infty }\left[p_{1}x+p_{2}\left(Q_{1}+Q_{2}-x\right)-g_{2}\left(y-\left(Q_{1}+Q_{2}-x\right)\right)\right]f\left(x,y\right)dy\;dx\\ -c_{1}Q_{1}-c_{2}Q_{2}-c\left(T\right)-w\left[k_{1}Q_{1}+\;\left(1-T\right)k_{2}Q_{2}-K\right]. \end{array}$$

When the green technology input *T* reaches to a certain level, it is obtained by solving the first partial derivatives of *Q*_1_ and *Q*_2_, respectively:

(30) $$\begin{array}{c} \frac{\partial \pi^{c}\left(Q_{1},Q_{2},T\right)}{\partial Q_{1}}=\left(r_{2}-r_{1}\right)F_{1}\left(Q_{1}\right)+\left(v_{1}-r_{2}\right)\int_{0}^{Q_{1}}\int_{0}^{Q_{1}+Q_{2}-x}f\left(x,y\right)\text{d}y\text{d}x+r_{1}-c_{1}-wk_{1},\\ \frac{\partial \pi^{c}\left(Q_{1},Q_{2},T\right)}{\partial Q_{2}}=\left(v_{1}-r_{2}\right)\left[\int_{0}^{Q_{1}}\int_{0}^{Q_{1}+Q_{2}-x}f\left(x,y\right)\text{d}y\text{d}x-F\left(Q_{1},Q_{2}\right)\right]+\left(v_{2}-r_{2}\right)F_{2}\left(Q_{2}\right)+r_{2}-c_{2}-w\left(1-T\right)k_{2}. \end{array}$$

It is obtained by solving the second partial derivatives of *Q*_1_ and *Q*_2_, respectively:

(31) $$\begin{array}{c} \frac{\partial^{2}\pi^{c}\left(Q_{1},Q_{2},T\right)}{\partial Q_{1}^{2}}=\left(r_{2}-r_{1}\right)f_{1}\left(Q_{1}\right)+\left(v_{1}-r_{2}\right)\left[\int_{0}^{Q_{1}}f\left(x,Q_{1}+Q_{2}-x\right)\text{d}x+\int_{0}^{Q_{2}}f\left(Q_{1},y\right)\text{d}y\right]<0,\\ \frac{\partial^{2}\pi^{c}\left(Q_{1},Q_{2},T\right)}{\partial Q_{2}^{2}}=\left(v_{1}-r_{2}\right)\left[\int_{0}^{Q_{1}}f\left(x,Q_{1}+Q_{2}-x\right)\text{d}x\int_{0}^{Q_{1}}f\left(x,Q_{2}\right)\text{d}x-\right]+\left(v_{2}-r_{2}\right)f_{2}\left(Q_{2}\right)\leq \left(v_{1}-r_{2}\right)\left[\int_{0}^{Q_{1}}f\left(x,Q_{1}+Q_{2}-x\right)\text{d}x+\int_{Q_{1}}^{\infty }f\left(x,Q_{2}\right)f\text{d}x\right]<0. \end{array}$$

The second mixed partial derivatives of *Q*_1_ and *Q*_2_ are solved as

(32) $$\begin{array}{c} \frac{\partial^{2}\pi^{c}\left(Q_{1},Q_{2},T\right)}{\partial Q_{1}\partial Q_{2}}=\frac{\partial^{2}\pi^{c}\left(Q_{1},Q_{2},T\right)}{\partial Q_{2}\partial Q_{1}}\\ =\left(v_{1}-r_{2}\right)\int_{0}^{Q_{1}}f\left(x,Q_{1}+Q_{2}-x\right)\text{d}x<0. \end{array}$$

Therefore,

(33) $$\begin{array}{c} \left|\begin{array}{cc} \frac{\partial^{2}\pi^{c}\left(Q_{1},Q_{2},T\right)}{\partial Q_{1}^{2}} & \frac{\partial^{2}\pi^{c}\left(Q_{1},Q_{2},T\right)}{\partial Q_{1}Q_{2}}\\ \frac{\partial^{2}\pi^{c}\left(Q_{1},Q_{2},T\right)}{\partial Q_{2}Q_{1}} & \frac{\partial^{2}\pi^{c}\left(Q_{1},Q_{2},T\right)}{\partial Q_{2}^{2}} \end{array}\right|=\left(v_{1}-p_{1}-r_{1}\right)f_{1}\left(Q_{1}\right)\left(v_{2}-p_{2}-r_{2}\right)f_{2}\left(Q_{2}\right)>0. \end{array}$$

Let *∂π*^*c*^(*Q*_1_, *Q*_2_, *T*)/*∂Q*_2_=0, it is obtained that

(34) $$\begin{array}{c} \frac{1}{k_{2}}\left(v_{1}-r_{2}\right)\left[\int_{0}^{Q_{1}}\int_{0}^{Q_{1}+Q_{2}-x}f\left(x,y\right)\text{d}y\text{d}x-F\left(Q_{1},Q_{2}\right)\right]+\left(v_{2}-r_{2}\right)F_{2}\left(Q_{2}\right)+r_{2}-c_{2}-w\left(1-T\right)k_{2}=0, \end{array}$$

*θ*_2_(*Q*_2_^*c*^)=(1 − *T*)*w* is obtained.

Let *∂π*^*c*^(*Q*_1_, *Q*_2_, *T*)/*∂Q*_1_=0 and *∂π*^*c*^(*Q*_1_, *Q*_2_, *T*)/*∂Q*_2_=0; it is obtained that

(35) $$\begin{array}{c} F_{1}\left(Q_{1}\right)+\frac{r_{2}-v_{1}}{r_{1}-r_{2}}\int_{0}^{Q_{1}}\int_{0}^{Q_{1}+Q_{2}-x}f\left(x,y\right)\text{d}y\text{d}x=\frac{r_{1}-c_{1}-wk_{1}}{r_{1}-r_{2}},\\ F_{2}\left(Q_{2}\right)+\frac{r_{2}-v_{1}}{r_{2}-v_{2}}\left[\int_{0}^{Q_{1}}\int_{0}^{Q_{1}+Q_{2}-x}f\left(x,y\right)\text{d}y\text{d}x-F\left(Q_{1},Q_{2}\right)\right]=\frac{r_{2}-c_{2}-\left(1-T\right)wk_{2}}{r_{2}-v_{2}}. \end{array}$$

The proof is justified.

Proposition 3 shows that under the regulation of carbon trade policy, considering downward substitution, manufacturing enterprises have an optimal green technology input level, and an optimal manufacturing decision, which maximizes manufacturing enterprise`s expected profit.

When *θ*_2_(*Q*_2_^*c*^) > (1 − *T*)*w*, under the regulation of carbon trade policy, considering downward substitution, manufacturing enterprises carry out green technology input, the marginal profit of producing the unit common product is higher than the price of the unit carbon emission rights. In this case, manufacturing enterprises will purchase carbon emission rights in the external carbon trade market. Manufacturing enterprises will increase the output of common products under the carbon trade policy until the marginal profit reaches (1 − *T*)*w*.

When *θ*_2_(*Q*_2_^*c*^) < (1 − *T*)*w*, under the regulation of carbon trade policy, considering downward substitution, manufacturing enterprises carry out green technology input, the marginal profit of producing the unit common product is less than the price of the unit carbon emission rights. In this case, manufacturing enterprises will sell carbon emission rights in the external carbon trade market.

When *θ*_2_(*Q*_2_^*c*^)=(1 − *T*)*w*, under the regulation of carbon trade policy, considering downward substitution, manufacturing enterprises carry out green technology input, the marginal profit of producing the unit common product is equal to the price of the unit carbon emission rights. In this case, manufacturing enterprises will not trade carbon emission rights in the external carbon trading market. At this time, under the carbon cap and trade policy, when manufacturing enterprise carries out green technology input, there is a manufacturing decision that maximizes the expected profit, which maximizes the enterprise`s expected profit.

In order to discuss the influence of green technology input on manufacturing decision of manufacturing enterprises under the regulation of carbon trade policy, considering downward substitution, the following proposition is obtained:

**Proposition 4 .**

*Q*_1_^*c*^ ≤ *Q*_1_^*e*^ < *Q*_1_^*∗*^*Q*_2_^*e*^ ≤ *Q*_2_^*c*^ < *Q*_2_^*∗*^

**Proof**

From Proposition 1, it is obtained that *θ*_1_(*Q*_1_) and *θ*_2_(*Q*_2_) is the decreasing function of *Q*_1_ and *Q*_2_, which *θ*_1_(*Q*_1_^*∗*^)=0, *θ*_2_(*Q*_2_^*∗*^)=0, *θ*_1_(*Q*_1_^*e*^)=*θ*_2_(*Q*_2_^*e*^)=*w*, *θ*_2_(*Q*_2_^*c*^)=(1 − *T*)*w*; therefore, *Q*_1_^*∗*^ > *Q*_1_^*e*^, *Q*_2_^*∗*^ > *Q*_2_^*c*^ ≥ *Q*_2_^*e*^.

Thus,

(36) $$\begin{array}{c} F_{1}\left(Q_{1}^{e}\right)=\frac{r_{1}-c_{1}-wk_{1}}{r_{1}-r_{2}}-\frac{r_{2}-v_{1}}{r_{1}-r_{2}}\int_{0}^{Q_{1}^{e}}\int_{0}^{Q_{1}^{e}+Q_{2}^{e}-x}f\left(x,y\right)\text{d}y\text{d}x\geq \frac{r_{1}-c_{1}-wk_{1}}{r_{1}-r_{2}}-\frac{r_{2}-v_{1}}{r_{1}-r_{2}}\int_{0}^{Q_{1}^{c}}\int_{0}^{Q_{1}^{c}+Q_{2}^{c}-x}f\left(x,y\right)\text{d}y\text{d}x=F_{1}\left(Q_{1}^{c}\right). \end{array}$$

And because *F*_1_(*·*) is an increasing function, so *Q*_1_^*c*^ ≤ *Q*_1_^*e*^.

Hence, the proof is justified.

Proposition 4 shows that under the regulation of carbon trade policy, considering downward substitution, the green technology input of manufacturing enterprises can improve the output of common products to a certain extent, but the output of green products will be reduced because of the weakening of downward substitution.

In order to further discuss the impact of carbon trade policy on manufacturing decision, the maximum expected profit of manufacturing enterprises under the carbon trade policy is as (37):

(37) $$\begin{array}{c} \pi^{c}\left(Q_{1}^{c},Q_{2}^{c}\right)=\pi^{n}\left(Q_{1}^{c},Q_{2}^{c}\right)-w\left[k_{1}Q_{1}^{c}+\left(1-T\right)k_{2}Q_{2}^{c}-K\right]-c\left(T\right). \end{array}$$

Let *H* be the difference value caused by the weakening of downward substitution after the green technology input of manufacturing enterprises under the carbon cap and trade policy.

Thus,

(38) $$\begin{array}{c} H=\pi^{e}\left(Q_{1}^{e},Q_{2}^{e}\right)-\pi^{c}\left(Q_{1}^{c},Q_{2}^{c}\right)\\ =\left(r_{2}-v_{1}\right)\left[\int_{0}^{Q_{1}^{c}}\int_{Q_{1}^{c}+Q_{2}^{c}-x}^{\infty }\left(Q_{1}^{c}-x\right)f\left(x,y\right)\text{d}y\text{d}x+\int_{0}^{Q_{1}^{c}}\int_{Q_{2}^{c}}^{Q_{1}^{c}+Q_{2}^{c}-x}\left(y-Q_{2}^{c}\right)f\left(x,y\right)\text{d}y\text{d}x+v_{1}\int_{0}^{Q_{1}^{c}}\int_{Q_{2}^{c}}^{Q_{1}^{c}+Q_{2}^{c}-x}\left(y-Q_{2}^{c}\right)f\left(x,y\right)\text{d}y\text{d}x+v_{1}\int_{0}^{Q_{1}^{c}}\int_{Q_{2}^{c}}^{\infty }\left(Q_{1}^{c}-x\right)f\left(x,y\right)\text{d}y\text{d}x+v_{2}\int_{Q_{1}^{c}}^{\infty }\int_{0}^{Q_{2}^{c}}\left(Q_{2}^{c}-y\right)f\left(x,y\right)\text{d}y\text{d}x\geq 0\right]. \end{array}$$

**Proposition 5 .**

under the regulation of carbon trade policy, considering downward substitution, when manufacturing enterprises carry out green technology input, there is an optimal strategy to make *π*^*c*^(*Q*_1_^*c*^, *Q*_2_^*c*^, *T*) ≥ *π*^*e*^(*Q*_1_^*e*^,*Q*_2_^*e*^) ≥ *π*^*a*^(*Q*_1_^*a*^, *Q*_2_^*a*^).

**Proof**

When *π*^*e*^(*Q*_1_,*Q*_2_) takes the maximum value, then *π*^*e*^(*Q*_1_^*e*^, *Q*_2_^*e*^) > *π*^*n*^(*Q*_1_^*∗*^, *Q*_2_^*∗*^) − *w*(*k*_1_*Q*_1_^*∗*^+*k*_2_*Q*_2_^*∗*^ − *K*). If *K* ≥ *k*_1_*Q*_1_^*∗*^+*k*_2_*Q*_2_^*∗*^,it is obtained from Proposition 1 that in this case, *π*^*a*^(*Q*_1_^*a*^, *Q*_2_^*a*^)=*π*^*n*^(*Q*_1_^*∗*^, *Q*_2_^*∗*^);therefore, $\pi^{e}\left(Q_{1}^{e},Q_{2}^{e}\right)-\pi^{a}\left(Q_{1}^{a},Q_{2}^{a}\right)>-w\left(\begin{array}{c} k_{1} \end{array}Q_{1}^{*}+k_{2}Q_{2}^{*}-K\right)>0$. Thus, *π*^*e*^(*Q*_1_^*e*^, *Q*_2_^*e*^) > *π*^*a*^(*Q*_1_^*a*^, *Q*_2_^*a*^).

If $K<\begin{array}{c} k_{1} \end{array}Q_{1}^{*}+k_{2}Q_{2}^{*}$, in this case, *K*=*k*_1_*Q*_1_^*a*^+*k*_2_*Q*_2_^*a*^,when *π*^*e*^(*Q*_1_, *Q*_2_) takes the maximum value, then *π*^*e*^(*Q*_1_^*e*^, *Q*_2_^*e*^) ≥ *π*^*n*^(*Q*_1_^*a*^, *Q*_2_^*a*^) − *w*(*k*_1_*Q*_1_^*a*^+*k*_2_*Q*_2_^*a*^ − *K*); it is obtained from Corollary 1 that *π*^*a*^(*Q*_1_^*a*^, *Q*_2_^*a*^)=*π*^*n*^(*Q*_1_^*a*^, *Q*_2_^*a*^);therefore, *π*^*e*^(*Q*_1_^*e*^,*Q*_2_^*e*^) − *π*^*a*^(*Q*_1_^*a*^,*Q*_2_^*a*^) ≥ −*w*(*k*_1_*Q*_1_^*a*^+*k*_2_*Q*_2_^*a*^ − *K*)=0. Thus, *π*^*e*^(*Q*_1_^*e*^, *Q*_2_^*e*^) ≥ *π*^*a*^(*Q*_1_^*a*^, *Q*_2_^*a*^). Given the above, *π*^*e*^(*Q*_1_^*e*^, *Q*_2_^*e*^) ≥ *π*^*a*^(*Q*_1_^*a*^, *Q*_2_^*a*^).

From (26) to (37), it is obtained that (*H* +*c*(*T*)+*wk*_2_[(1 − *T*)(*a*_2_ − *b*_2_*p*_2_^*c*^+*z*_1_^*c*^) − (*a*_2_ − *b*_2_*p*_2_^*e*^+*z*_1_^*e*^)]*π*^*c*^(*Q*_1_^*c*^, *Q*_2_^*c*^) − *π*^*e*^(*Q*_1_^*e*^, *Q*_2_^*e*^)=−*H* − *c*(*T*) − *wk*_2_[(1 − *T*)*Q*_2_^*c*^  − *k*_2_*Q*_2_^*e*^]).

1. When *H*+*c*(*T*)+*wk*_2_[(1 − *T*)*k*_2_*Q*_2_^*c*^ − *k*_2_*Q*_2_^*e*^] < 0, *π*^*e*^(*Q*_1_^*e*^, *Q*_2_^*e*^) − *π*^*c*^(*p*_1_^*c*^, *p*_2_^*c*^, *T*)=*H*+*c*(*T*)+*wk*_2_[(1 − *T*)*k*_2_*Q*_2_^*c*^ − *k*_2_*Q*_2_^*e*^] < 0. At this point, *π*^*c*^(*p*_1_^*c*^, *p*_2_^*c*^, *T*) < *π*^*e*^(*p*_1_^*e*^, *p*_2_^*e*^). Therefore, carrying out green technology input at this point will increase enterprises expected profit.
2. When *H*+*c*(*T*)+*wk*_2_[(1 − *T*)*k*_2_*Q*_2_^*c*^ − *k*_2_*Q*_2_^*e*^]=0, *π*^*e*^(*Q*_1_^*e*^, *Q*_2_^*e*^) − *π*^*c*^(*p*_1_^*c*^, *p*_2_^*c*^, *T*)=*H*+*c*(*T*)+*wk*_2_[(1  − *T*)*k*_2_*Q*_2_^*c*^ − *k*_2_*Q*_2_^*e*^]=0. At this point, *π*^*c*^(*p*_1_^*c*^, *p*_2_^*c*^, *T*)=*π*^*e*^(*p*_1_^*e*^, *p*_2_^*e*^). Therefore, carrying out green technology input at this point will not increase enterprise`s expected profit, thus manufacturing enterprises abandonment green technology input rationally.
3. When *H*+*c*(*T*)+*wk*_2_[(1 − *T*)*k*_2_*Q*_2_^*c*^ − *k*_2_*Q*_2_^*e*^] > 0, *π*^*e*^(*Q*_1_^*e*^,*Q*_2_^*e*^) − *π*^*c*^(*p*_1_^*c*^,*p*_2_^*c*^,*T*)=*H*+*c*(*T*)+*wk*_2_[(1 − *T*)*k*_2_*Q*_2_^*c*^ − *k*_2_*Q*_2_^*e*^] > 0. At this point, *π*^*c*^(*p*_1_^*c*^,*p*_2_^*c*^,*T*) > *π*^*e*^(*p*_1_^*e*^,*p*_2_^*e*^). Therefore, carrying out green technology input at this point will increase enterprises expected profit.

Therefore, *π*^*c*^(*Q*_1_^*c*^,*Q*_2_^*c*^,*T*) ≥ *π*^*e*^(*Q*_1_^*e*^, *Q*_2_^*e*^) ≥ *π*^*a*^(*Q*_1_^*a*^, *Q*_2_^*a*^). Hence, the proof is justified.

Proposition 5 shows that under the regulation of carbon trade policy, considering downward substitution, the appropriate green technology input to common products will increase the expected profit of manufacturing enterprises. On the other hand, under the carbon trade policy, while the carbon emission rights are saved, the downward substitution will be weakened after enterprises investing in green technology, and the size between them determines whether manufacturing enterprises carry out green technology input or not.

## 4. Conclusion

Based on the stochastic market demand of products, this paper studies the low-carbon manufacturing decision considering downward substitution and green technology input under the regulation of carbon trade policy, which provides an idea for the operation and management of manufacturing enterprises under the background and pressure of carbon emission reduction. The analysis shows that the government's carbon trade policy will have a great impact on the manufacturing decisions of manufacturing enterprises, and the output of common products and green products of manufacturing enterprises will be no higher than the optimal output without carbon emission reduction policy. Therefore, manufacturing enterprises must attach importance to the regulation of government carbon emission reduction policies. Further analysis shows that manufacturing enterprises can respond to the constraints of carbon reduction policies through strategies such as output adjustment, carbon trade, or green technology input. Thus, green technology input of manufacturing enterprises can increase the expected profit level to a certain extent. However, when manufacturing enterprises invest in green technology, they should consider the size between difference value caused by the weakening of downward substitution and the carbon emission rights saved after green technology input. For while green technology input is good for the environment, it is not always good for manufacturing enterprises. Therefore, the government should consider both the environment and enterprises to formulate carbon emission reduction policies that are beneficial to both of them, so that the enterprise is more inclined to adopt green technology. In this way, government will promote “win-win” for both enterprise development and environmental protection so as to promote the maximization of social welfare. In conclusion, due to the increasingly severe environmental deterioration, the government's carbon emission reduction policies and regulations will become more and more stringent in the future. With the enhancement of public environmental awareness, consumers will also have higher demand for green low-carbon products. Thus, the green and low-carbon products are the inevitable direction for manufacturing enterprises. Therefore, in order to gain the competitive advantage, strategies of low-carbon productions such as green technology input are the inevitable choice for manufacturing enterprises in the future.

## Data Availability

All data used in this study can be accessed by request.
